# Factors associated with trajectories of women’s quality of life and association with substance use 12 months post-treatment

**DOI:** 10.1186/1940-0640-10-S1-A47

**Published:** 2015-02-20

**Authors:** Hyunyong Park, Elizabeth M Tracy, Meeyoung O Min, Lenore Kola, Alexandre Laudet

**Affiliations:** 1 Jack, Joseph and Morton Mandel School of Applied Social Sciences, Cleveland, OH, 44106, USA; 2 National Development and Research Institutes, Inc., New York, NY, 10010

## Background

Quality of life (QOL) has become an increasingly recognized component of recovery for women with substance use disorders (SUDs) [1-3]. However, little is known about different trajectories of QOL among women with SUD over time. This study: a) identified heterogeneous QOL trajectories; b) examined predictors related to QOL trajectories; and c) investigated the association between QOL trajectories and substance use 12 months post-treatment intake.

## Methods

Data were collected from 377 women at three county-funded treatment programs. Women were interviewed at 1 week, and at 1, 6, and 12 months post-treatment intake (81% retention). The World Health Organization Quality of Life Measure (abbreviated version) assessed QOL over time. Latent Class Growth Model (LCGM) was performed to identify QOL trajectories of underlying subgroups of the women. The model fit was evaluated by the Bayesian Information Criteria and the sample sizes of the smallest class. Multinomial logistic regression explored demographic, clinical, and personal network factors associated with the QOL trajectories. Lastly, we explored the relationship between the QOL trajectories and substance use over 12 months.

## Results

The majority of participants were African American (60%), received government assistance (72.5%), and had co-occurring mental and substance use disorders (73.4%); 42 percent used alcohol and/or drugs during the 12 month follow-up period. Mean QOL at intake was 61.2 (SD = 15.62).

**Figure 1:**
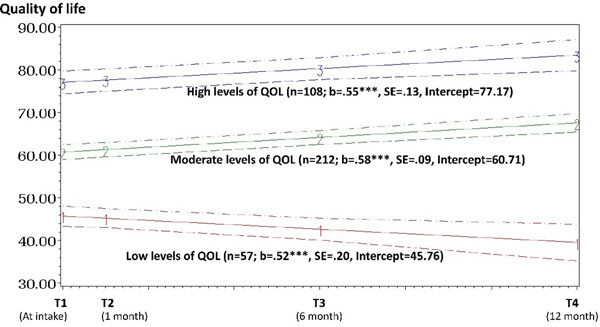
QOL trajectories among women with SUDs

LCGM indicated three different trajectories in QOL (Figure 1): a) consistently high QOL (n = 108; 28.6%); b) consistently moderate QOL (n = 212; 56.2%); and c) consistently decreasing QOL (n = 57; 15.1%). Multinomial logistic regression results (Table 1) shows that higher levels of trauma symptoms were associated with increased odds of belonging to the decreasing QOL group compared to either high QOL group or the moderate QOL group. Higher levels of abstinence self-efficacy and greater number of close network members were associated with decreased odds of belonging to the decreasing QOL group. Compared to the moderate QOL group, a greater number of critical network members were associated with increased odds of belonging to the decreasing QOL group. The decreasing QOL group was more likely to use substances at 12 months post-treatment intake (OR = 3.48; 95% CI = 1.62, 7.50) compared to the high QOL group.

**Table 1:** Results of multinomial logistic regression

	Decreasing QOL vs. High QOL (ref)	Moderate QOL vs. High QOL (ref)	Decreasing QOL vs. Moderate QOL (ref)
	
	*OR* (95% *CI*)	*OR* (95% *CI*)	*OR* (95% *CI*)
Treatment Modality	1.73 (0.65, 4.59)	0.78 (0.41, 1.49)	2.22 (0.98, 5.04)
Unstable Housing	2.41 (0.84, 6.85)	1.27 (0.57, 2.82)	1.90 (0.85, 4.25)
Dual Disorder	0.99 (0.34, 2.92)	1.07 (0.58, 1.99)	0.93 (0.35, 2.45)
Trauma Symptoms	**1.08 (1.06, 1.11)**	**1.03 (1.02, 1.05)**	**1.05 (1.03, 1.07)**
Abstinence Self-Efficacy	**0.97 (0.95, 0.99)**	**0.98 (0.96, 0.99)**	0.99 (0.98, 1.01)
Substance using Alters	1.01 (0.92, 1.11)	1.05 (0.99, 1.12)	0.97 (0.89, 1.04)
Concrete Support	0.95 (0.88, 1.03)	1.00 (0.95, 1.05)	0.95 (0.89, 1.02)
Sobriety Support	0.98 (0.89, 1.08)	1.03 (0.97, 1.11)	0.95 (0.88, 1.03)
Reciprocal Relationship	0.93 (0.84, 1.03)	0.97 (0.92, 1.04)	0.96 (0.88, 1.04)
Very Close Alters	**0.86 (0.78, 0.94)**	**0.93 (0.88, 0.98)**	0.92 (0.85, 1.002)
Critical Alters	1.04 (0.95, 1.13)	0.95 (0.89, 1.01)	**1.09 (1.004, 1.18)**

*Note.* Boldface indicates statistical significance at alpha level of .05.

## Conclusions and implications

Findings highlight three distinctive QOL trajectories and their association with substance use treatment outcome. Fifteen percent of the women had consistently low levels of QOL, which decreased over time and increased odds of substance use post-treatment, suggesting this group may need targeted intervention and follow-up.
